# A New Algorithm for Medical Color Images Encryption Using Chaotic Systems

**DOI:** 10.3390/e21060577

**Published:** 2019-06-10

**Authors:** Seyed Shahabeddin Moafimadani, Yucheng Chen, Chunming Tang

**Affiliations:** School of Mathematics and Information Science, Guangzhou University, Guangzhou 510006, China

**Keywords:** image encryption, medical color images, RGB, chaotic system

## Abstract

In this paper, we present a new algorithm based on chaotic systems to protect medical images against attacks. The proposed algorithm has two main parts: A high-speed permutation process and adaptive diffusion. After the implementation of the algorithm in the MATLAB software, it is observed that the algorithm is effective and appropriate. Also, to quantitatively evaluate the uniformity of the histogram, the chi-square test is done. Key sensitivity analysis demonstrates that images cannot be decrypted whenever a small change happens in the key, which indicates that the algorithm is suitable. Clearly, part of special images is selected to test the selected plain-text, like an all-white image and an all-black image. Entropy results obtained from the implementation of the algorithm on this type of images show that the proposed method is suitable for this particular type of images. In addition, the obtained results from noise and occlusion attacks analysis show that the proposed algorithm can withstand against these types of attacks. Moreover, it can be seen that the images after encryption and decryption are of good quality; the measures such as the correlation coefficients, the entropy, the number of pixel change rate (NPCR), and the uniform average change intensity (UACI) have suitable values; and the method is better than previous methods.

## 1. Introduction

The confidentiality of patient information is one of the vital security aspects of electronic health services. For example, the confidentiality of patients’ medical records is necessary. In addition, the methods of protection should be improved due to the rapid advancement of technologies for accessing the personal information of individuals. The security and privacy of medical image transferring is one of the acute subjects that should be seriously considered in telecare medical information systems (TMIS). In the past years, medical images were grayscale, but today, color images have entered the medical arena, and they can show more accurate information about body conditions. Color images that are acquired by new scanners using the Medipix3RX chip technology are very important in the medical arena. Image-data transferring from a position to another via an unsafe network are usually determined in qualifications of privacy, validity, totality, and confidentiality. Therefore, more significance should be given to the security of the sensitive data that are included in medical images by DICOM (digital imaging communication in medicine). In this textuality, many problems using various cryptographic techniques have been proposed in the literature to overcome this problem [1,2,3,4]. In their paper, Abdel-Nabi and Al-Haj proposed a hybrid encryption algorithm using watermarking that offers high embedding capabilities for medical images. The proposed algorithm is a combination of reversible data-caching techniques with standard encryption techniques for ensuring the security requirements for transferred and stored medical images [5]. In their paper, Abdmouleh et al. presented a partial cryptographic approach that was based on the digital wavelet transform (DWT) and was JPEG2000 compliant to ensure the safe transfer and storage of medical images [6]. Lakshmi et al. presented a similar algorithm using a discrete wavelet transform (DWT), with the difference being that they used a fuzzy chaotic map for the watermarking [7]. In their paper, Cao et al. presented a medical image encryption algorithm using edge maps that were derived from a source image. The algorithm consists of three parts: Bit-plane decomposition, a random-sequence generator, and permutation [8]. Ismail et al. presented the double-humped (DH) logistic map to produce pseudorandom numbers keys (PRNG) in their paper. The generalized parameter that is added to the map provides more control on the map chaotic range [9]. Jeong et al. proposed a new medical image encrypting method using a 2D chaotic map and C-MLCA in their article. The 2D chaotic map is a construction with self- guarding attributes, which moves the location of the pixel and encrypts the image [10]. In their method, Ke et al. offered an encryption algorithm that was based on reversible data using the MSB-based prediction [11]. In their study, Nematzadeh et al. proposed an encryption method for medical images based on a hybrid pattern of the improved genetic algorithm (IGA) and paired map lattices. First, the assumed way employs a paired map lattice to produce a some of secure cipher-images as a primitive population of the IGA. Then, it exerts the IGA to both increase the entropy of the cipher images and reduce the algorithm’s calculations time [12]. In their paper, Singh et al. proposed a medical image encryption scheme algorithm using the improved ElGamal encryption technique. Their proposed method made a new contribution since the necessity for separate calculations for encoding plain messages to elliptic curve coordinates was removed. The algorithm using the improved version of the ElGamal encryption scheme is designed to encrypt medical images [13]. Suganya and Amudha’s proposed method uses two encryption algorithms, namely, RC4 and AES, which are the stream cipher and block cipher algorithms, respectively. The main objective of this method is to provide integrity control for medical images, although they are encrypted. Experimental security analysis is conducted using 8-bit ultrasound images and 16-bit positron emission tomography (PET) images [14]. The chaos systems that have become popular today have been used in many types of research. For example: Liang and Qi investigated mechanical analysis of generates the Chen chaotic system to the extensile Kolmogorov system. In Hu et al.’s research, the Chen chaotic system is designed as a pseudorandom sequence producer. In their research, Wang et al. used the memristor chaotic systems (MCSs). Gong et al. provided a method for image encryption based on hyper-chaotic system and discrete fractional random transform. Michail et al.’s research was based on chaotic systems and hash functions to implement totally self-checking (TSC). James et al., in their research based on chaotic systems and hash functions, discussed the performance of SHA-3 256- bit core. Ahmad and Das, based on chaos and hash algorithms, discussed Hardware performance analysis of SHA-256 and SHA-512 algorithms on FPGAs. In their research, Xu et al. improved chaotic cryptosystem based on circular bit shift and XOR operations. Pareek and Patidar, in their research, designed medical image protection based on genetic algorithm and chaotic system. Hua et al., in their study, designed medical image encryption using hash algorithm high-speed scrambling [15,16,17,18,19,20,21,22,23,24]. Also, Chai et al. designed a color image encryption method based on dynamic DNA encryption and chaotic system [25] and Ma et al. provided a new method of plaintext-related and chaos-based image encryption [26]. Niyat et al. offered an image encryption algorithm with the rule of cellular automata (CA). CA is a self- establishing construction with a group of cells in which any cell is updated based on certain regulation that are to depend on a limited number of neighboring cells [27]. Chen et al. designed an image encryption method based on hyperchaotic system in the turner transfigure domain. The RGB ingredients of the main color image are encrypted into 1D circulation. [28]. The method in [5] focused on the embedding capacity, but no results are given with respect to other criteria, such as the correlation coefficients entropy, the number of pixel change rate (NPCR), or the uniform average change intensity (UACI). The methods in [6,7] are based on the digital wavelet transform (DWT) and allow for safe transport, but they do not provide suitable results with respect to the correlation coefficients. The results that were obtained in [8] are only suitable for the NPCR, and other values are not suitable. In the method in [9], the values that are obtained are appropriate, but they are only suitable for grayscale images. The results of the methods in [10,11,12] are only suitable with respect to their correlation coefficients, but there are no improvements with respect other criteria. The results that are obtained in [13,14] are only suitable with respect to entropy. The methods in [27,28] perform well in color images encrypting and can be used to encrypt medical images. Nevertheless, our method is better in terms of the correlation coefficients, the entropy, the NPCR, and the UACI.

Our aim in this paper is to provide an algorithm that protects medical color images based on chaotic systems and SHA-256 systems. The algorithm is composed of two parts: A high-speed permutation process and adaptive diffusion. For this reason, in Section 2, we will present the basic concepts of chaotic systems and SHA-256 systems. In Section 3, we will describe the proposed algorithm’s equations, and in Section 4, the empirical results from the implementation of the proposed algorithm that is simulated in the MATLAB software will be given. In Section 5, we compare the results of the proposed method with previous methods, and in Section 6, we will explain the quality and appropriateness of this method.

## 2. Preliminary Work

### 2.1. Chaotic Systems

Chaos theory is a chapter of mathematics centralization on the action of dynamical systems that are highly sensitive to initial situation. “Chaos” is a notion denoting that within the obvious accidentalness of chaotic systems, there are basically models, stable feedback rings, iteration, self- likeness, fractals, self- formation, and dependence on programming at the initial part, which is known to have sensitive to depend on initial situation.

Little differences in initial situation, like those owing to rounding errors in numerical calculations, output widely in different outcomes for dynamical systems, thus, generally interpretation of the long-term oracle of their action impossible. This action is known as certain chaos, or simply chaos. The theory was tabloid by Edward Lorenz as follows:

Chaos: When the design specifies the future, but the proximate present does not proximately specify the future. 

In 1963, Lorenz studied chaotic systems using a nonlinear differential equation, which is one of the first examples of algebraic chaotic systems in dissipative systems. Chaotic systems are very abundant in nature and they are used in many branches of science, such as the physics of dynamics and photonics, medical sciences, chemistry, and demography. In recent years, much research has been done on chaotic systems by scientists and more practical systems have been introduced, such as Chen’s system, Lu’s system, and Qi’s 3D four-wing chaotic system [15,16,17]. The chaotic system that is used in this algorithm is the Chen-based hyper-chaotic system. The Chen-based hyper-chaotic system in [18] is described as follows:

(1) $$\{\begin{array}{c} \begin{array}{c} \dot{s}=a\left(t-s\right)\\ \dot{t}=\mathrm{ds}-\mathrm{su}+\mathrm{ct}-v \end{array}\\ \begin{array}{c} \dot{u}\;=\;\mathrm{st}-\mathrm{bu}\\ \dot{v}\;=\;s+w \end{array} \end{array}$$

where s, t, u, and v are the fixed variables and a, b, c, d, and w are the controlling parameters of the system. The dynamical cycle will be hyper-chaotic when a = 36, b = 3, c = 28, d = −16, and −0.7 < w < 0.7. 

### 2.2. SHA-256 (Secure Hash Algorithm 256)

A cryptographic hash (sometimes called a “digest”) is a type of ‘signature’ for a text or data file. The SHA-256 products an almost-unique 256-bit (32-byte) signature for a text. The SHA-256 is a type of the deputy hash functions to the SHA-1 (referential to as SHA-2) and is one of the existing powerfulness hash functions. The SHA-256 is not much more complicated to code than the SHA-1 and has not yet been agreement in any path. The 256-bit key makes it a good common-function for the AES. It is explained in the NIST (National Institute of Standards and Technology) standard ‘FIPS 180-4’. The NIST also prepared a number of test vectors to investigate the validity of its execution [19,20,21].

To the SHA-256, the message is decomposed to n blocks with 512 bits, and at the end of its the final block, bit ‘1’ is added to be followed by k zero bits, where k is the least nonnegative the answer path of the equation l + 1 + k = 448mod512. Next, a 64-bit binary block that is equivalent to l is added. Clearly, a “1” followed by k “0” s that is followed by 64 bits are added at the end of M to generate a crooked message of length 512∗n bits. For instance, the 8-bit ASCII message “abc” has a length of l = 8 × 3 = 24. Therefore, the message is padded with a one bit, then 448 − (24 + 1) = 423 zero, and then the 64 bits of the length of message ${\left(11000\right)}_{2}$ = ${\left(24\right)}_{10}$. Then, one message plan carries out on the blocks of M, generating the $W_{t}$ amount, any of which is to the corresponding t-th repetition of the transmutation. The transmutation takes $W_{t}$, a fixed value, $K_{t}$ and the primary amounts $H^{\left(0\right)}$ (in the repetition one) or the values generated in the past repetition; carries out the transmutation procedure; and produces a series of hash values via a number of repetitions. The last produced hash value is considered as the message digest, h [19]. The SHA-256 needs 64 repetition to generate its message digest. The round contains additives and rational functions that are set to generate the round’s output values. The included NLFs are shown in Equation (2):

(2) $$Ch\left(x,\;y,z\right)=\left(x•y\right)⊕\left(\bar{x}•y\right)\;Maj\left(x,\;y,\;z\right)=\left(x•y\right)⊕\left(x•z\right)⊕\left(x•z\right)\;\overset{256}{\underset{0}{\sum }}\left(x\right)=ROTR^{2}\left(x\right)⊕ROTR^{13}\left(x\right)⊕ROTR^{22}\left(x\right)\;\overset{256}{\underset{1}{\sum }}\left(x\right)=ROTR^{6}\left(x\right)⊕ROTR^{11}\left(x\right)⊕ROTR^{25}\left(x\right)$$

where ⊕, •, and ¯ denote the XOR, AND, and NOT bitwise rational functions, respectively, and *x*, *y*, and *z* are 32-bit words. $ROTR^{X}$ Shows x right round bit spin. According to the 64 $W_{t}$ values that are necessary, the first 16 are organized by the 512-bit input block whereas the remaining 48 $W_{t}$ amounts are calculated using Equation (3). The functions $\sigma_{0}$ and $\sigma_{1}$ are computed using Equation (4).

(3) $$W_{t}=\sigma_{1}^{\left\{256\right\}}\left(W_{t-2}\right)+W_{t-7}+\sigma_{0}^{\left\{256\right\}}\left(W_{t-15}\right)+W_{t-16}\;\;16\leq t\leq 63$$

(4) $$\sigma_{0}^{\left\{256\right\}}=ROTR^{7}\left(x\right)⊕ROTR^{18}\left(x\right)⊕SHR^{3}\left(x\right)\;\sigma_{1}^{\left\{256\right\}}=ROTR^{17}\left(x\right)⊕ROTR^{19}\left(x\right)⊕SHR^{10}\left(x\right)$$

**Figure 1:**
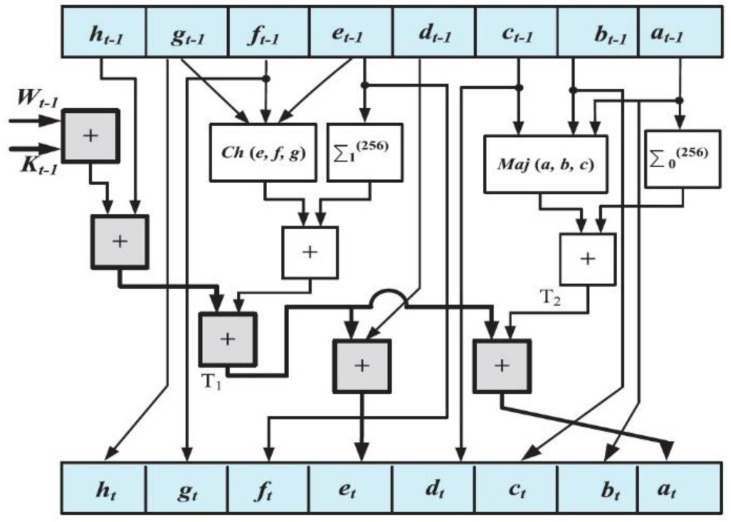
Secure hash algorithm 256 (SHA-256) base transformation rounds.

Here, $SHR^{x}$ stands for the right bit shift. The SHA-256 base transformation rounds are shown in Figure 1.

### 2.3. SHA-256 Architectures

The performance of the SHA-256 construction’s transformation round is shown in Figure 1. It takes, as inputs, eight 32-bit characters, ($a_{t-1}-h_{t-1}$), the value $W_{t-1}$, and the stable value $K_{t-1}$, performs the calculations shown in Figure 1, and generates the values ($a_{t}-h_{t}$) after 64 repetitions [19]. 

## 3. Proposed Algorithm

The proposed algorithm has two parts: A high-speed permutation process and adaptive diffusion. 

### 3.1. High-Speed Permutation Process

Step 1. First, the plain image P of size M×N×D is used as the input; it has the initial state values of *a*0, *b*0, *c*0, and *d*0; and uses the SHA-256 function, which is constructed according to the plain image. We consider DM=D×M and s = sum(sha256)/(64×256), which reshape matrix P into the 2D matrix P1. Then, the new values of the chaos system *a*0, *b*0, *c*0, and *d*0 are generated using Equation (4) as follows:

(5) $$\{\begin{array}{c} \begin{array}{c} a0=s+a0-⎣s+a0⎦\\ b0=s+b0-⎣s+b0⎦ \end{array}\\ \begin{array}{c} c0=s+c0-⎣s+c0⎦\\ d0=s+d0-⎣s+d0⎦ \end{array} \end{array}.$$

Step 2. Then, the initial values and parameters are used to iterate the chaotic systems to obtain the vectors *a*1, *a*2, *a*3, and *a*4 and quantize to generate four different vectors *PR*1, *PC*1, *PR*2, and *PC*2, which are as follows:

(6) $$\{\begin{array}{c} \begin{array}{c} PR1=\left(\left|a1\right|-⎣a1⎦\right)\times 10^{15}\;mod\;N+1\\ PC1=\left(\left|a2\right|-⎣a2⎦\right)\times 10^{15}\;mod\;DM+1 \end{array}\\ \begin{array}{c} PR2=\left(\left|\;a3\right|-⎣a3⎦\right)\times 10^{15}\;mod\;N+1\\ PC2=\left(\left|\;a4\right|-⎣a4⎦\right)\times 10^{15}\;mod\;DM+1 \end{array} \end{array}.$$

Here, we have to use the circshift (shift array circularly) rule and its definition is as follows: If A and B are matrixes, B = circshift(A, shiftsize) circularly shifts the values in the array A using the shift size elements [22]. 

Step 3. We consider $PR,PC\in {\mathbb{N}}^{DM\times N}$ and for i=1 to DM, if i is odd, then we get the following:

(7) $$\{\begin{array}{c} PR\left(:,i\right)=circshift\;P1\left(i,:\right)\;by\;step\;PR1\left(i\right)\\ and\;else\\ PR\left(:,i\right)=circshift\;P1\left(i,:\right)by\;step-PR2\left(i\right) \end{array}.$$

Step 4. For *j* = 1 to *N*, if *j* is odd, then we get the following:

(8) $$\{\begin{array}{c} PC\left(:,j\right)=circshift\;IR\left(:,j\right)\;by\;step\;IC1\left(j\right)\\ and\;else\\ PC\left(:,j\right)=circshift\;IR\left(:,j\right)by\;step-IC2\left(j\right) \end{array}.$$

Now, *P*2 = *PC* is the permutated image.

This Process explained in Algorithm 1 with the title: High-speed Permutation Process. 

**Algorithm 1** High-speed Permutation Process  Input: Image P of size M × N × D, Initial state: a0, b0, c0, d0, and Sha256 value of P  Output: Permutated Image Let DM = D × M, s = sum (sha256)/(64 × 256), and reshape P to 2-dimension matrix P1.Generate a new initial value of the chaotic system: a0, b0, c0, d0;Use the initial value and parameters to iterate the chaotic system to get the vectors: a1, a2, a3, a4, and quantize to generate four different vectors: PR1, PC1, PR2, PC2.Set PR, PC ∈N^DM^×^N^for i = 1 to DM doif i is odd then PR(:, i) = circshift P1(i,:) by step PR1(i)else PR(:, i) = circshift P1(i,:) by step −PR2(i) end ifend forfor j = 1 to N do if j is odd then PC(:,j) = circshift PR(:,j) by step PC1(j)      else PC(:,j) = circshift PR(:,j) by step −PC2(j)end ifend forLet PC be the permutated imagereturn Permutated Image

### 3.2. Adaptive Diffusion

Step 1. First, the other initial values and parameters are used to iterate the chaotic systems to obtain the vectors *a*110, *b*110, *c*110, and *d*110, and they are quantized to generate four different vectors *a*11, *b*11, *c*11, and *d*11.

We set *N*0 as a random number, N00=N0+1 and nn=(DM×N)/2.

(9) $$\{\begin{array}{c} \begin{array}{c} a11=\;⎣|⎣\left(a110+b110\right)\times 10^{15})⎦|mod2^{3}⎦+1\\ \;b11=\;⎣|⎣\left(b110-a110\right)\times 10^{15})⎦|mod2^{3}⎦+1 \end{array}\\ \begin{array}{c} c11=\;⎣|⎣\left(c110+d110\right)\times 10^{15})⎦|mod2^{8}⎦\\ d11=\;⎣|⎣\left(c110+d110\right)\times 10^{15})⎦|mod2^{8}⎦ \end{array} \end{array}$$

Step 2. Set $A,B\in {\mathbb{N}}^{DM\times N}$ and i=1 to DM. If i≥1 and i≤ DM/2, then we get the following:

(10) $$\{\begin{array}{c} A\left(i,:\right)=a11\left(\left(\left(i-1\right)\times N+1\right):\left(i\times N\right),:\right)\\ B\left(i,:\right)=c11\left(\left(\left(i-1\right)\times N+1\right):\left(i\times N\right),:\right) \end{array}.$$

Otherwise,

(11) $$\{\begin{array}{c} A\left(i,:\right)=b11\left(\left(\left(i-1-\left(DM/2\right)\right)\times N+1\right):\left(\left(i-\left(DM/2\right)\right)\times N\right),:\right)\\ B\left(i,:\right)=d11\left(\left(\left(i-1-\left(DM/2\right)\right)\times N+1\right):\left(\left(i-\left(DM/2\right)\right)\times N\right),:\right) \end{array}.\;$$

Step 3. Here, we have to apply *bitcircshift* rule, which is an action that is done on all of the bits of a binary amount, in which they are transformed by a determined number of locations to the left or right. *Bitcircshift* is used when the operand is being used as a series of bits relatively than generally. In other words, the operand behaves as single bits that show something and are not values [22].

Let P2=PC, and $P3\in {\mathbb{N}}^{DM\times N}$. If i=1 to DM and j=1 to N, then we get the following:

(12) P3(i,j)=bitcircshift P2(i,j)by step A(i,j).

Step 4. Let $key_{r0}={\left(a110\left(1:N\right)+b110\left(1:N\right)\right)}^{’}/2$ and $key_{c0}=\left(c110\left(1\;:\;DM\right)+d110\left(1\;:\;DM\right)\right)/2$, and quantize them as $key_{r}$ and $key_{c}$, respectively, in [0, 255].

(13) $$\{\begin{array}{c} key_{r}=⎣|key_{r0}|\times 10^{15}⎦mod256\\ key_{c}=⎣|key_{c0}|\times 10^{15}⎦mod256 \end{array}$$

Step 5. Set $P4R,\;P4C\in {\mathbb{N}}^{DM\times N}$. If i=1 to DM, r1=circshift(B(i,;),[0,i]) and i=1, then we get the following:

(14) $$\{\begin{array}{c} P4R\left(i,:\right)=\left(\left(P3\left(i,:\right)+r1\right)mod256\right)⊕key_{r}\\ and\;else\\ P4R\left(i,:\right)=\left(\left(P3\left(i,:\right)+r1\right)mod256\right)⊕P4R\left(\left(i-1\right),:\right) \end{array}.$$

Step 6. If j=1 to N, c1=circshift(B(j,:),[j,0]) and j=1, then we get the following:

(15) $$\{\begin{array}{c} \;P4C\left(:,j\right)=\left(\left(P4R\left(:,j\right)+c1\right)mod256\right)⊕key_{c}\\ and\;else\\ \;P4C\left(:,j\right)=\left(\left(P4R\left(:,j\right)+c1\right)mod256\right)⊕P4C\left(:,\left(j-1\right)\right) \end{array}\;\;.$$

P4C, the final image, is encrypted.

This Process explained in Algorithm 2 with the title: Adaptive diffusion.

**Algorithm 2** Adaptive diffusion  Input: Input data from permutation procession  Output: Encrypted Image 1:Use other initial values and parameters to iterate the chaotic system again to get the vectors:a110, b110, c110, d110, and quantize them to generate four different vectors: a11, b11, c11, d11.2:Set A, B ∈N^DM^×^N^3:for i = 1 to DM do4: if i ≥ 1 and i ≤ DM/2 then5:  A(i,:) = a11(((i − 1) × N + 1) : (i × N),:)6:  B(i,:) = c11(((i − 1) × N + 1) : (i × N),:)7:else8:    A(i,:) = b11(((i − 1 − (DM/2)) × N + 1) : ((i − (DM/2)) × N),:) B(i,:) = d11(((i − 1 − (DM/2)) × N + 1) : ((i − (DM/2)) × N),:)9:end if10:end for11:Let P2 = PC, and set P3 ∈N^DM^×^N^12:for i = 1 to DM do13:for j = 1 to N do14:P3(i,j) = bitcircshift P2(i,j) by step A(i,j)15:end for16:end for17:Let key_r0 = (a110(1 : N)+b110(1 : N))′/2, key_c0 = (c110(1 : DM)+d110(1 : DM))/2, and quantize them key_r, key_c, in [0, 255]18:Set P4R, P4C ∈N^DM^×^N^19:for i = 1 to DM do20:r1 = circshift (B(i,;),[0,i])21:if i = 1 then22:  P4R(i,:) = bitxor(mod((P3(i, :)+r1), 256), key_r)23:else24:  P4R(i,:) = bitxor(mod((P3(i, :)+r1), 256), P4R((i-1), :))25:     end if26:end for27:for j = 1 to N do28:c1 = circshift (B(j,:),[j,0])29:if j = 1 then30:  P4C(:,j) = bitxor(mod((P4R(:, j)+c1), 256), key_c)31:else32:  P4C(:,j) = bitxor(mod((P4R(:, j)+c1), 256), P4C(:, (j-1)))33:end if34:end for35:Let P4C be the final encrypted image36:return Encrypted Image  The decryption process is inverse encryption process.  Input: Input data from permutation procession  Output: Encrypted Image

**Figure 2:**
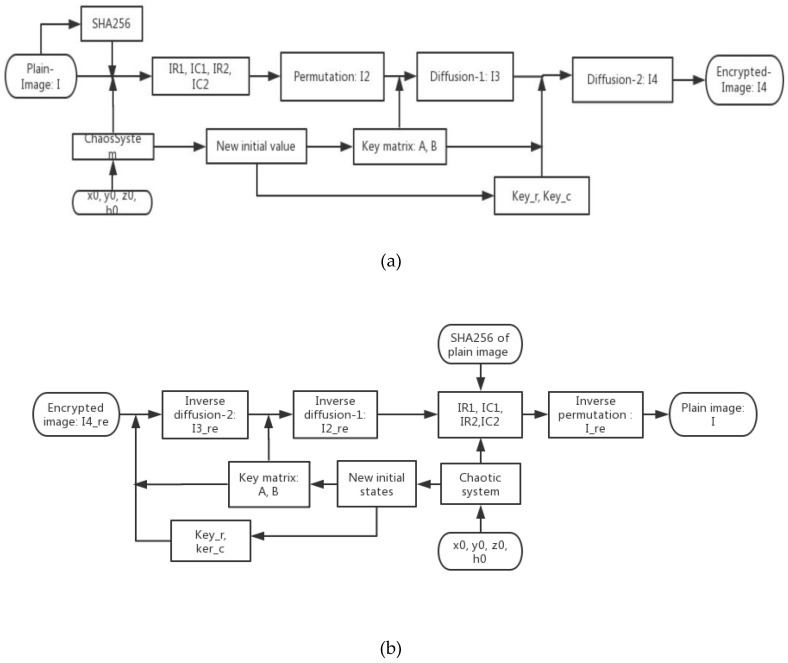
Schematic of the proposed encryption algorithm (**a**), and schematic of the proposed decryption algorithm (**b**).

Our proposed algorithm is a symmetric algorithm. The decryption procedure is the opposite of the encryption method and decryption is done using the encryption method’s formulas. This is shown in Figure 2.

**Remark** **1.** *The proposed algorithm is suitable for all color images (RGB). Because medical images are very important in today’s technological world, we decided to use the proposed algorithm for medical images.*

## 4. Experiment Result and Security Analysis

In this section, we implemented the proposed algorithm on two medical color images using the MATLAB 2017a software environment (in personal computer with core i7, 3.4GHz, RAM 16GB). As we stated in the introduction, the medical color images are obtained using the Medipix3RX chip technology that is used in today’s imaging devices [1,2,3,4].

**Figure 3:**
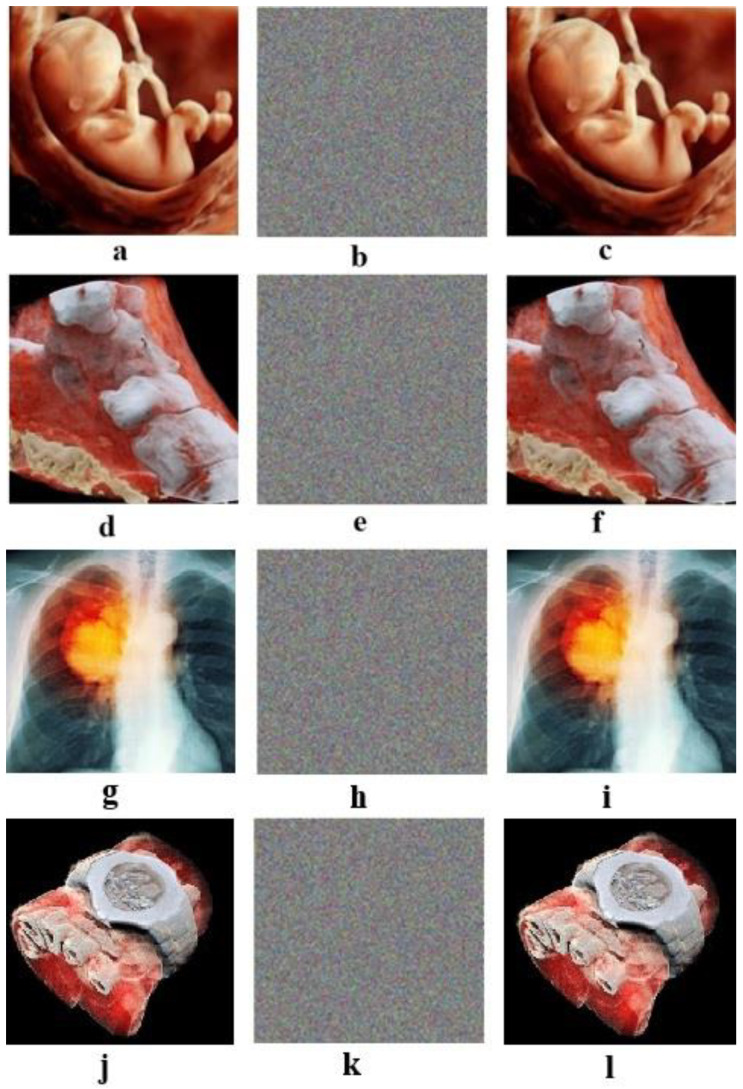
(**a**,**d**,**g**,**j**): Plain images. (**b**,**e**,**h**,**k**): Respective encrypted images. (**c**,**f**,**i**,**l**): Respective decrypted images. Initial values for all images: (a0 = 0.1314, b0 = 0.5214, c0 = 0.3698, and d0 = 0.8419). Values of the chaotic system: Image (**a**): sha256 = ‘9ADBBFB88CFD90C23CE114E47402054E6DDC4182510E80980EA7151CD11E6D18’, image (**d**): sha256 = ‘8BF6A886E4B58D2B530749EE9BAB54A3C360D406DC5B901CC169D7870FA3CA09’, image (**g**): sha256 = ‘49A22186DB65786789CD1391CDE4D9737039E758F39A45C59D8338DE05353337’, and image (**j**): sha256 = ‘6EB1ADE45F27A67E09A25265835F05BC11E057255DA81359299631F4724936C8’.

For example, four color images 256 × 256 in size have been selected as the plain images. In Figure 3, images b, e, h, and k are images that are encrypted by the proposed algorithm for the plain images a, d, g, and j, respectively; and images c, f, i, and l are the decrypted images.

### 4.1. Security Analysis

As seen, it is not possible to visually compare the plain images with images that were obtained from the decryption process, and the measures such as the correlation coefficients of two adjacent pixels in the plain image and the cipher image, the entropy, the NPCR (number of pixel change rate), and the UACI (uniform average change intensity) should be mathematically examined. We consider an example of a baby’s image.

### 4.2. Histogram Analysis

**Figure 4:**
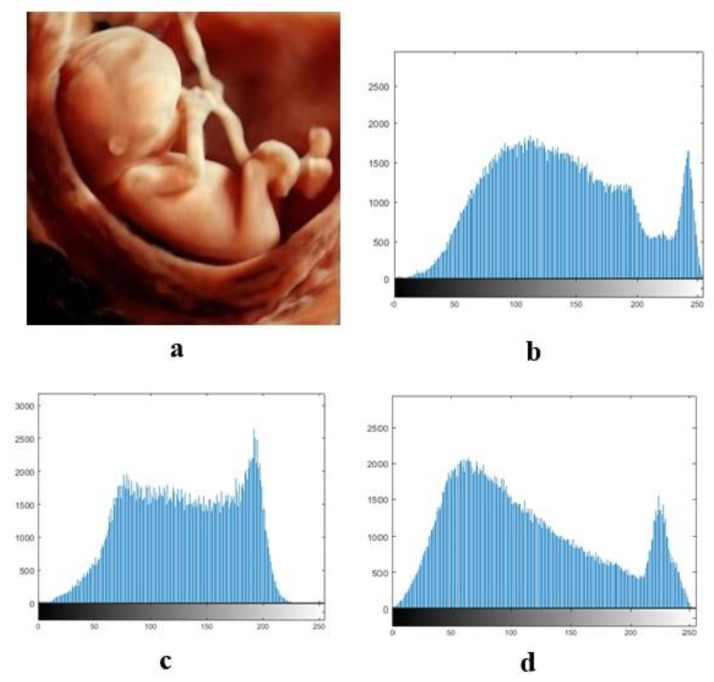
(**a**): Plain image baby, and (**b**–**d**): R, G, and B histograms, respectively.

Color images include three main color channels (red, green, and blue), and these images are called RGB images. Figure 4 shows the histograms of these three channels that are observed for the baby’s image.

**Figure 5:**
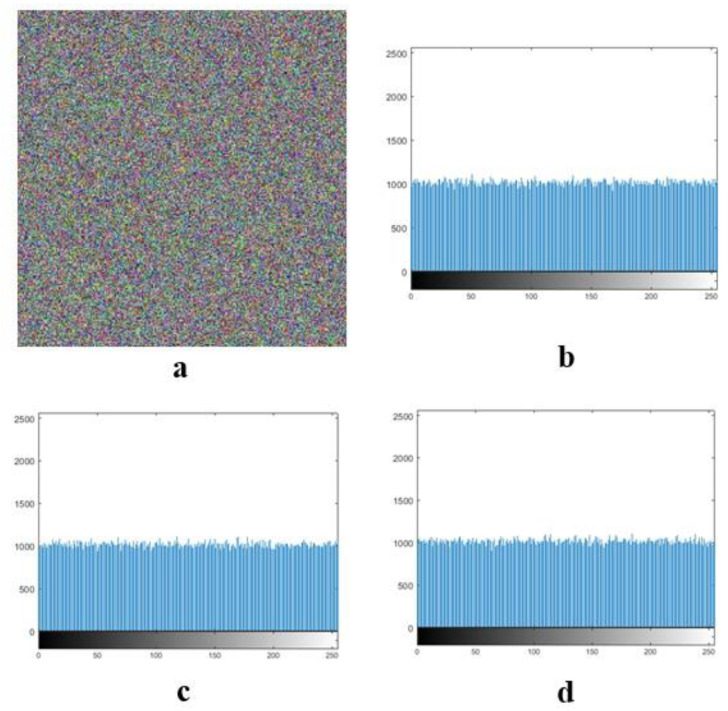
(**a**): Baby’s decrypted image, and (**b**–**d**): R, G, and B histograms, respectively.

In Figure 5, we can see the baby’s decrypted image from the three-channel color histograms.

Chi-square Analysis 

Statistical analysis is a type of the commonplace cryptology procedures. The monotony of the histogram of cipher demonstrates the strength of the encryption path to statistical analysis. The ocular effect of the histogram is not sufficient to verify the accident of a cipher image’s pixel values [26]. To quantitatively measure the monotony of the histogram, we use the chi-square test as a metric. The description of the chi-square is as follows:

(16) $$\chi_{exp}^{2}=\overset{Q}{\underset{i=1}{\sum }}\frac{{\left(Q_{i}-e_{i}\right)}^{2}}{e_{i}},\;e_{i}=\frac{M\times N}{Q},$$

where *Q* = 256 in our method, $o_{i}$ is the observed incidence frequency of each rate on the histogram of the ciphered image, $e_{i}$ is the envisage incidence frequency of the uniform distribution, and M×N is the length of an image trail. For an ideal image encryption system, the empirical chi-square value should be less than the theoretic amount. With the importance level of 0.05, the theoretic chi-square value is 293 [26]. The chi-square test conclusions and transition rates are listed in Table 1 and Table 2. All the test images transition the test, which shows that our plan has a satisfying encryption effect.

### 4.3. Correlation Analysis

**Figure 6:**
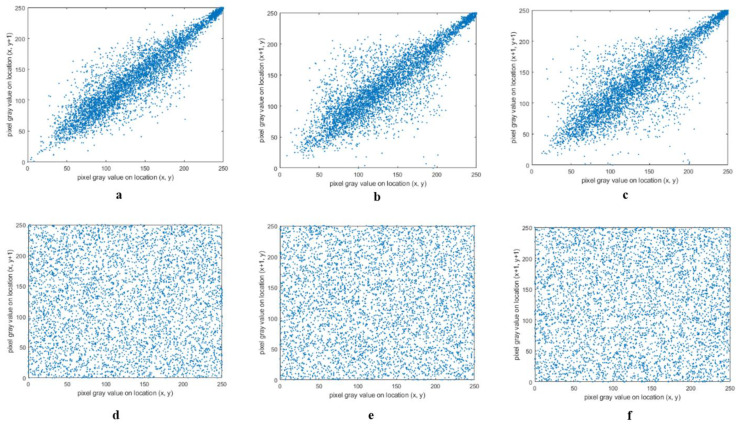
Correlation histograms. (**a**–**c**): For the plain image; and (**d**–**f**): For the cipher image.

The correlation coefficient of two adjacent pixels in the plain image and the cipher image is one of the important factors in determining the quality of image encryption algorithms [23]. In Figure 6, we can see the correlation histograms for the plain image and cipher image, and the correlation histograms are shown in three directions: Horizontal, vertical, and diagonal.

In Table 3, the numerical values of the correlation for the plain image and the cipher image are given, and the values in the table are calculated for three directions: Horizontal, vertical, and diagonal. We can specifically see that the correlation coefficients of the plain image are near to 1, however the correlation coefficients of the cipher-image are about equal 0, which may explain why the designed encrypted algorithm has a powerful resistance to possible statistical attacks. The table specifies that the proposed algorithm has the required quality. The correlation coefficient of two adjacent pixels in the plain image and the cipher image is obtained as follows:

(17) $$r_{xy}=\frac{E(\left(x_{i}-E\left(x\right)\right)\left(y_{i}-E\left(y\right)\right)}{\sqrt{D\left(x\right)D\left(y\right)}}$$

where

$$E\left(x\right)=\frac{1}{N}\sum_{i=1}^{n}x_{i},\;\;D\left(x\right)=\frac{1}{N}\sum_{i=1}^{n}{\left(x_{i}-E\left(x\right)\right)}^{2}\;.$$

$E(\left(x_{i}-E\left(x\right)\right)\left(y_{i}-E\left(y\right)\right)=cov\left(x,\;y\right)$, $E\left(x\right)=\frac{1}{N}\overset{N}{\underset{i=1}{\sum }}x_{i}$ is the expected value, *N* is the number of image pixels, and $D\left(x\right)=\frac{1}{N}\overset{N}{\underset{i=1}{\sum }}{\left(x_{i}-E\left(x\right)\right)}^{2}$ is the variance. *x* and *y* are the gray values of two adjacent pixels, and *N* is the total number of pixels that are chosen from the image.

### 4.4. Entropy Analysis

The entropy randomly measures the data sequence and is defined as follows [24]:

(18) $$H\left(S\right)=\overset{2^{N}-1}{\underset{i=0}{\sum }}P\left(s_{i}\right)\log\left(\frac{1}{P\left(s_{i}\right)}\right)$$

where *N* is the number of grayscale levels in an image, and $P\left(s_{i}\right)$ is the incidence possibility of grayscale “I” in the image. The entropy amount will be 8 for images that are wholly accidentally generated. The nearer the entropy of an encryption method is to 8, the less foreseeable it is, and thus, the more secure the plan. The entropies for the designed encryption method have been measured for a sample image and the conclusions are shown in Table 4.

### 4.5. NPCR (Number of Pixel Change Rate) and UACI (Uniform Average Change Intensity)

In a differential attack, a little variation is built to the plain image, and the designed algorithm is employed to encrypt the plain image before and after this variation. These two encrypted images have been evaluated to detect any possible connection between the plain image and the cipher image. The (UACI) and the (NPCR) are two indicator that are regularly used by researchers to test the differential attack resistor of any image encryption method [12]. 

Suppose that $C_{1}$ and $C_{2}$ are two cipher images that are encrypted from two plain images with only one-bit difference. The NPCR and UACI are defined as follows:

(19) $$NPCR\left(C_{1},C_{2}\right)=\sum_{i,j}\frac{H\left(i,j\right)}{M}\times 100\%$$

and

(20) $$UACI\left(C_{1},C_{2}\right)=\underset{i,j}{\sum }\frac{\left|C_{1}\left(i,j\right)-C_{2}\left(i,j\right)\right|}{\left(S-1\right)\times M}\times 100\%$$

where *M* shows the total number of pixels in any cipher-image, S illustrates the number of allowed pixels, and H(i,j) demonstrates the difference between $C_{1}$ and $C_{2}$, which is specified as follows.

(21) $$H\left(i,j\right)=\{\begin{array}{c} \begin{array}{ccc} 0, & if & C_{1}\left(i,j\right)=C_{2}\left(i,j\right) \end{array}\\ \begin{array}{ccc} 1, & if & C_{1}\left(i,j\right)\neq C_{2}\left(i,j\right) \end{array} \end{array}.$$

The larger the NPCR and UACI are, the better the quality of the algorithm. For four randomly selected points, the NPCRs and UACIs are listed in Table 5.

### 4.6. Key Space

The key space for encryption algorithms should be large enough to withstand potential attacks. The minimum key space should be $2^{100}$. The input values of ($x_{0},y_{0},z_{0},h_{0},SHA256$) act as a secret key, and, based on this, the secret key space is $10^{14}\times 10^{14}\times 10^{14}\times 10^{14}\times 2^{128}=10^{56}\times 2^{128}$. This indicates that the designed method has good key space. 

### 4.7. Key Sensibility Analysis

**Figure 7:**
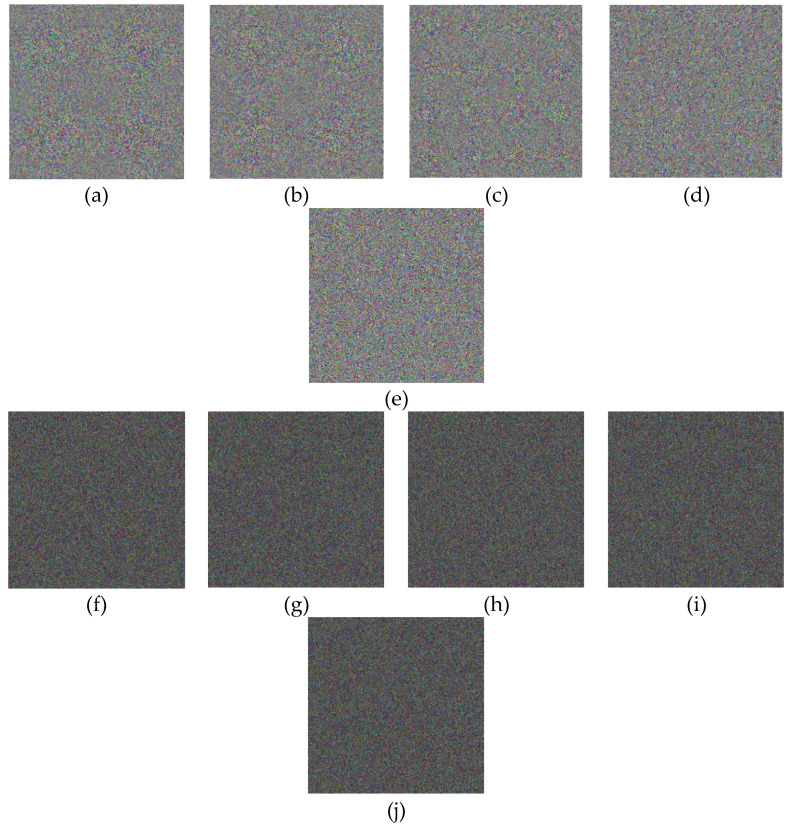
Cipher-images within authentic primary keys and the differences between them and the plain encrypted images: (**a**–**e**) Five new encrypted images with the keys $x_{0}$ + $10^{-14}$, $y_{0}$ + $10^{-14}$, $z_{0}$ + $10^{-14}$, $h_{0}$ + $10^{-14}$, and SHA256+$2^{-128}$, respectively; and (**f**–**j**) differences between the unauthentic encrypted images and the plain image.

A safe encryption system must be sensitive to the key; for example, the little change of encryption keys can lead to very different cipher image, and a small change of the decryption keys cannot decrypt the image. Several key sensitivity tests are performed. Figure 7 shows the encrypted images of the baby (plain image b). Figure 3a shows the encrypted image using user keys with a 1-bit difference. The plain encrypted image is indicated in Figure 3b. When the keys of the initial state $x_{0},y_{0},z_{0},h_{0},\;\mathrm{and}\;SHA256$ are changed by one bit (i.e., $10^{-14}$ for $x_{0},y_{0},z_{0},\;\mathrm{and}\;h_{0}$ and $2^{-128}$ for SHA256), the five new encrypted images are obtained and shown in Figure 7a–e. We compare them with the image in Figure 7e, and the five differential images are shown in Figure 7f–j. This shows that there are very big differences between the images in Figure 7e,f–j.

In addition, to experience the capability of the designed method to resist the cipher text attack, the keys $x_{0},y_{0},z_{0},\mathrm{and}\;h_{0}$ will be modified by $10{\;}^{-14}$ and SHA256 will be changed by $2^{-128}$ to decrypt the plain encrypted image. The decrypted images are indicated in Figure 7, which are wholly different from the plain image. Therefore, it can be seen that the cipher text cannot be suitably decrypted without the correct keys, which shows that the proposed method can effectively hamper the cipher text merely attack.

### 4.8. Known-Plain Image and Chosen-Plain Image Analysis

**Figure 8:**
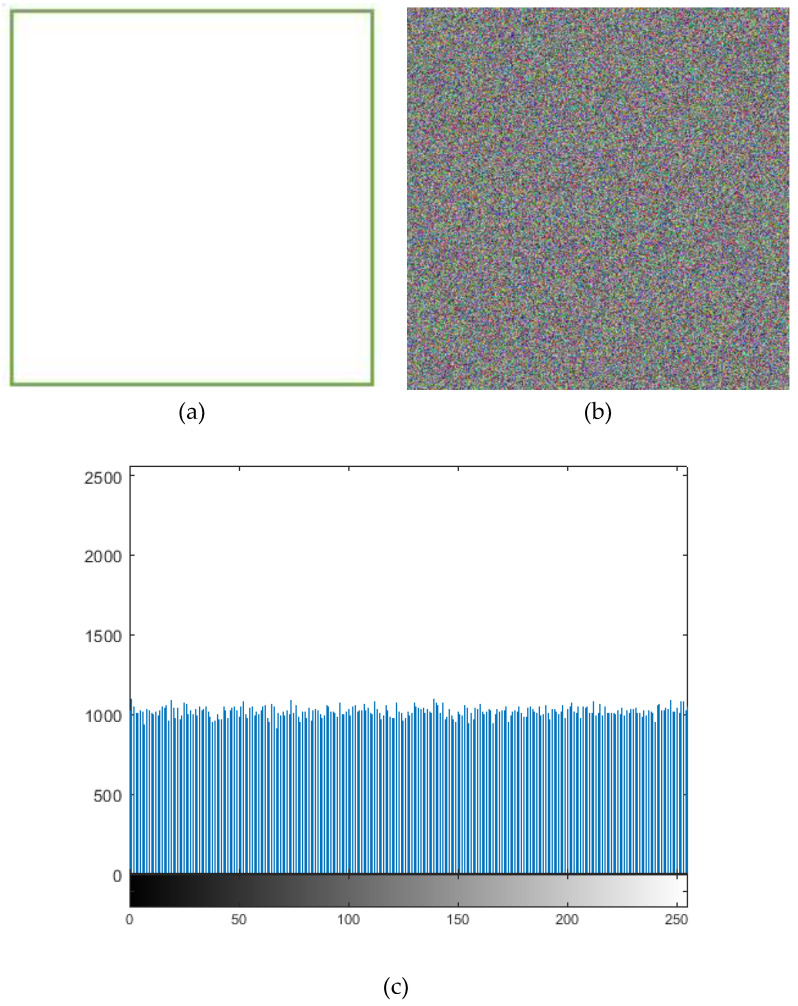

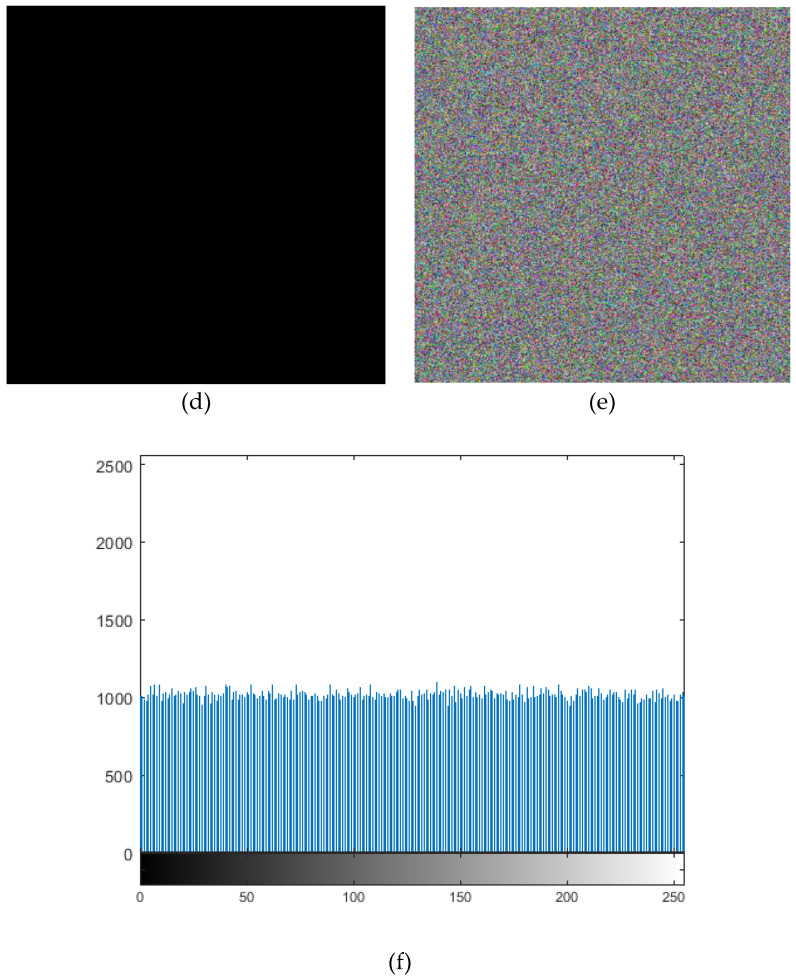
Selected plain-image test for white and black images, display (**a**) the full-white image, (**b**) the cipher image of panel (**a**), (**c**) the histogram of channel R (**b**), (**d**) the full-black image, (**e**) the cipher image of panel (**d**), and (**f**) the histogram of channel R.

Clearly, some specific images are selected to test the selected plain-text attack, like a full-white image in Figure 8a and a full-black image in Figure 8d. The results are shown in Figure 8, which indicate that the cryptology is appropriate for these specific images and can resist the chosen-plain-text attack. In Table 6, we can see the entropy values for all-white and all-black images. It can be seen that all the values that are obtained are close to 8, indicating the suitability of the proposed algorithm.

### 4.9. Noise Attack and Occlusion Attack

**Figure 9:**
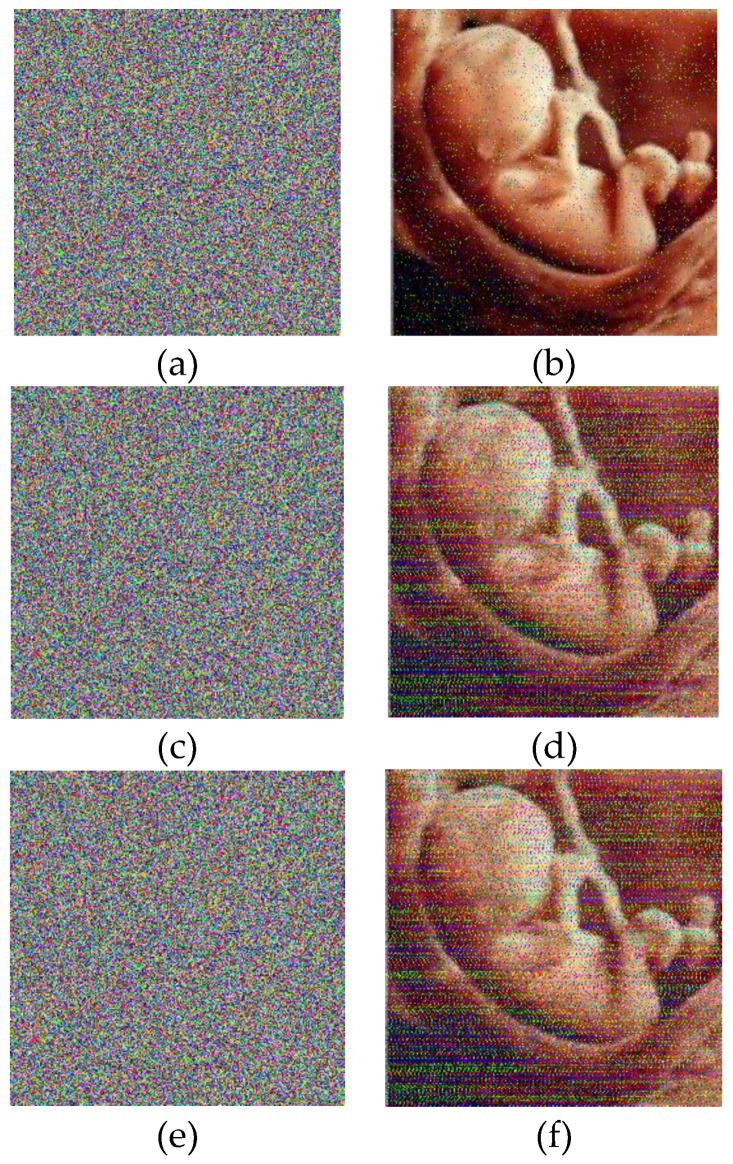
Noise attack test results. (**a**,**b**) Gaussian noise (GN); (**c**,**d**) salt and pepper noise (SPN); (**e**,**f**) speckle noise (SN).

**Figure 10:**
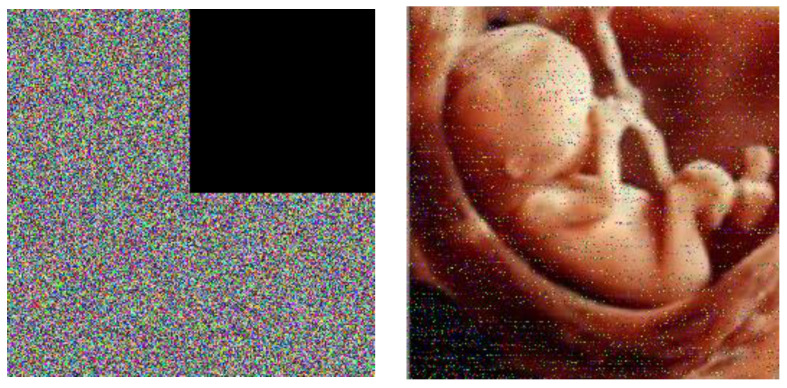

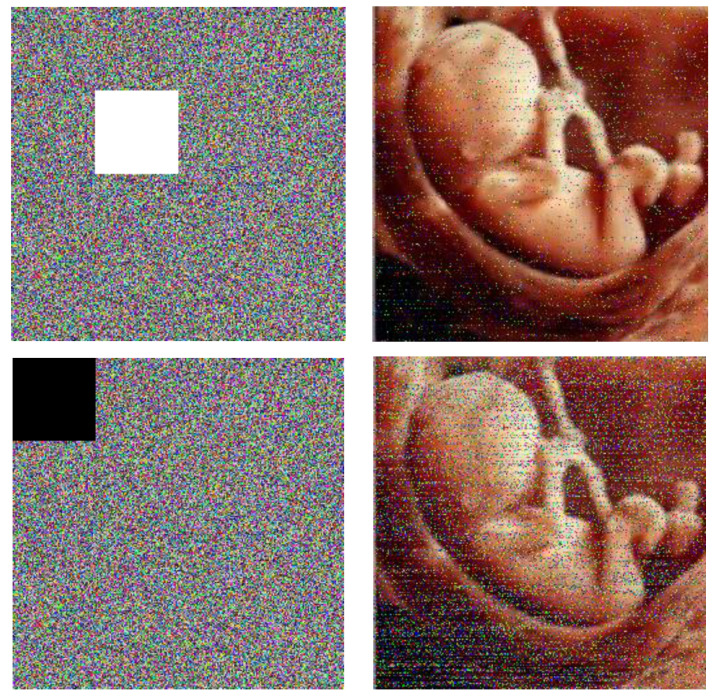
Occlude attack test results.

In the digital world, the images will unexpectedly experience noise and occlude attacks in the transition process, and an effective cryptology must be strong against them. The baby image is used as the test image. Figure 9 shows the noisy cipher images that are contaminated by Gaussian noise (GN), salt and pepper noise (SPN), and speckle noise (SN) with different noise compression and their decrypted images. As seen from Figure 10, the most information of the plain image can be intuitively identified from the decrypted image’s presentation of the cipher images with different occlusion effects and their corresponding retrieved images. Specifically, the decrypted images still include most date of the baby image. The PSNR (peak signal-to-noise-ratio) is employed to compute the condition of the decrypted image after a possible attack. For a gray image, the PSNR and MSE can be computed as follows:

(22) $$PSNR=10\;\times log_{10}\left(\frac{255\times 255}{MSE}\right)\left(db\right)$$

(23) $$MSE=\frac{1}{mn}\overset{m}{\underset{i=1}{\sum }}\overset{n}{\underset{j=1}{\sum }}{\left|\left|I_{1}\left(i,j\right)-I_{2}\left(i,j\right)\right|\right|}^{2}MSE=\frac{1}{mn}\overset{m}{\underset{i=1}{\sum }}\overset{n}{\underset{j=1}{\sum }}{\left|\left|I_{1}\left(i,j\right)-I_{2}\left(i,j\right)\right|\right|}^{2}$$

where MSE shows the mean square error between the cipher image $I_{1}\left(i,j\right)$ and the plain image $I_{2}\left(i,j\right)$, and *m* and *n* are the width and height, respectively [25]. The results are explained in Table 7 and Table 8.

## 5. Comparison

In this section, we will compare the results that were obtained from the proposed algorithm with previous algorithms. The results that were obtained from the designed method are measured with the methods in References [10,24] with respect to their correlation, entropy, NPCR, and UACI, and the key space was compared with references [9,12,24]. The results of the comparison are shown in Table 9, Table 10, Table 11, Table 12, Table 13 and Table 14.

## 6. Conclusions

In this paper, we present a new algorithm based on chaotic systems to protect these images against attacks. The proposed algorithm has two main parts: A high-speed permutation process and adaptive diffusion, which lead to a very efficient and reliable approach in this regard. By examining the results that were obtained from the implementation of the proposed algorithm in the MATLAB software environment and comparing these results with existing methods, it is observed that the designed method is better than those algorithms with respect to the important factors that are mentioned. Such that, to quantitatively evaluate the uniformity of the histogram, the chi-square test is done and the obtained results are desirable. Also, key sensitivity analysis shows that the image is not decrypted with a small change in the key, which indicates that the algorithm is suitable. Clearly, particular images are selected to experiment the selected plain-text, such as a full-white image and a full-black image. Entropy results obtained from the implementation of the algorithm on this type of images show that the proposed method is suitable for this particular type of images. In the real world, the images will inevitably experience noise and occlude attacks while shifting, and an effective cryptosystem must be powerful versus them. The obtained results from noise and occlusion attacks analyses show that the proposed algorithm can withstand against these types of attacks. It is also observed that the values that are obtained with respect to the entropy, NPCR, and UACI are better than those from the methods in existing papers. As we have already mentioned, the key space should be large enough (at least 2^100). Compared to the old methods, we observe that the key space of our method is very large and more resistant than other methods in dealing with attacks. 

## Figures and Tables

**Table 1:** Chi-square test results (part 1).

Images	a			d		
Channels	R	G	B	R	G	B
$x_{test}^{2}$	247.0762	204.9082	220.0742	251.8379	260.2695	256.4063
$x_{255.0.05}^{2}$	293	293	293	293	293	293
Pass or not	Yes	Yes	Yes	Yes	Yes	Yes

**Table 2:** Chi-square test results (part 2).

Images	g			j		
Channels	R	G	B	R	G	B
$x_{test}^{2}$	285.8359	261.9980	247.2793	250.0836	210.7622	231.1039
$x_{255.0.05}^{2}$	293	293	293	293	293	293
Passor not	Yes	Yes	Yes	Yes	Yes	Yes

**Table 3:** Correlation coefficients in the plain image and the cipher image.

Image	Channel	Plain-Text			Cipher-Text		
		H	V	D	H	V	D
a	R	0.9952	0.9978	0.9897	–0.0115	0.0048	–0.0026
	G	0.9825	0.9881	0.9688	0.0109	0.0097	–0.0161
	B	0.9833	0.9803	0.9615	–0.0224	–0.0091	0.0062
d	R	0.9217	0.8673	0.8580	0.0097	–0.0091	–0.0094
	G	0.8575	0.7647	0.7328	0.0015	0.0075	–0.0055
	B	0.9140	0.8966	0.8626	0.0137	0.0051	0.0065
g	R	0.9942	0.9968	0.9887	–0.0125	0.0047	–0.0025
	G	0.9815	0.9871	0.9678	0.0109	0.0095	–0.0160
	B	0.9823	0.9793	0.9605	–0.0214	–0.0093	0.0063
j	R	0.9217	0.8663	0.8570	0.0095	–0.0092	–0.0093
	G	0.8575	0.7637	0.7318	0.0014	0.0073	–0.0056
	B	0.9140	0.8956	0.8616	0.0135	0.0052	0.0066

**Table 4:** Information entropies results for plain and cipher images.

Image	Channel	Image a	Image d	Image g	Image j
Plain image	R	6.9581	7.7047	6.9571	7.7067
	G	6.8945	7.4724	6.8955	7.4734
	B	6.1365	7.7502	6.1355	7.7512
	RGB	7.2528	7.7604	7.2548	7.7614
Cipher image	R	7.9992	7.9991	7.9982	7.9993
	G	7.9993	7.9991	7.9983	7.9995
	B	7.9993	7.9991	7.9994	7.9981
	RGB	7.9997	7.9996	7.9996	7.9994

**Table 5:** Number of pixel change rates (NPCRs) and uniform average change intensities (UACIs) of different positions (%).

Position	(12,34)	(34,56)	(56,78)	(78,90)
NPCR	99.6232	99.6215	99.6170	99.5971
UACI	33.4574	33.4952	33.5326	33.4755

**Table 6:** Information entropy of the full-black image and full-white image.

Image	R	G	B
Black	7.9993	7.9994	7.9993
White	7.9994	7.9994	7.9993

**Table 7:** Noise attack test results.

Item	PSNR		
	R	G	B
Salt and Pepper	34.2863	33.6124	33.3711
Gaussian	30.6104	29.9219	29.7102
Speckle	30.6023	29.8951	29.7059

**Table 8:** Occlude attack test results.

Item	PSNR		
	R	G	B
Salt and pepper	34.0961	33.6237	33.5174
Gaussian	34.0106	33.6064	33.4199
Speckle	31.0996	30.6760	30.4795

**Table 9:** Correlation coefficients results in the original image and the encrypted image for the proposed method and the two methods that were presented in existing methods.

Image	Methods	Channel	Plain Image			Cipher Image		
			H	V	D	H	V	D
Image a	proposed	R	0.9952	0.9978	0.9897	–0.0115	0.0048	–0.0026
		G	0.9825	0.9881	0.9688	0.0109	0.0097	–0.0161
		B	0.9833	0.9803	0.9615	–0.0224	–0.0091	0.0062
	Algorithm [10]	R	0.9952	0.9978	0.9897	–0.0122	–0.0117	–0.0238
		G	0.9825	0.9881	0.9688	–0.0113	0.0079	–0.0230
		B	0.9833	0.9803	0.9615	–0.0099	0.0149	0.0092
	Algorithm [24]	R	0.9952	0.9978	0.9897	–0.0114	–0.0110	–0.0174
		G	0.9825	0.9881	0.9688	–0.0206	–0.0071	0.0180
		B	0.9833	0.9803	0.9615	–0.0134	–0.0106	–0.00194
Image d	proposed	R	0.9217	0.8673	0.8580	0.0097	–0.0091	–0.0094
		G	0.8575	0.7647	0.7328	0.0015	0.0075	–0.0055
		B	0.9140	0.8966	0.8626	0.0137	0.0051	0.0065
	Algorithm [10]	R	0.9217	0.8673	0.8580	0.0133	–0.0095	–0.0070
		G	0.8575	0.7647	0.7328	–0.0182	0.0230	–0.0056
		B	0.9140	0.8966	0.8626	–0.0282	–7.3230 × 10^–4^	–0.0073
	Algorithm [24]	R	0.9217	0.8673	0.8580	–0.0154	–0.0242	0.0094
		G	0.8575	0.7647	0.7328	–0.0059	0.0109	1.7711 × 10^–4^
		B	0.9140	0.8966	0.8626	–0.0216	–0.0089	0.0077
Image g	proposed	R	0.9942	0.9968	0.9887	–0.0125	0.0047	–0.0025
		G	0.9815	0.9871	0.9678	0.0109	0.0095	–0.0160
		B	0.9823	0.9793	0.9605	–0.0214	–0.0093	0.0063
	Algorithm [10]	R	0.9942	0.9968	0.9887	–0.0136	0.0066	–0.0035
		G	0.9815	0.9871	0.9678	0.0113	0.0103	–0.0190
		B	0.9823	0.9793	0.9605	–0.0237	–0.0098	0.0073
	Algorithm [24]	R	0.9942	0.9968	0.9887	–0.0129	0.0049	–0.0076
		G	0.9815	0.9871	0.9678	0.0111	0.0101	–0.0171
		B	0.9823	0.9793	0.9605	–0.0231	–0.0156	0.0067
Image j	proposed	R	0.9217	0.8663	0.8570	0.0095	–0.0092	–0.0093
		G	0.8575	0.7637	0.7318	0.0014	0.0073	–0.0056
		B	0.9140	0.8956	0.8616	0.0135	0.0052	0.0066
	Algorithm [10]	R	0.9217	0.8663	0.8570	0.0115	–0.0102	–0.0105
		G	0.8575	0.7637	0.7318	0.0084	0.0094	–0.0083
		B	0.9140	0.8956	0.8616	0.0196	0.0067	0.0089
	Algorithm [24]	R	0.9217	0.8663	0.8570	0.0115	–0.0111	–0.0106
		G	0.8575	0.7637	0.7318	0.0082	0.0103	–0.0074
		B	0.9140	0.8956	0.8616	0.0161	0.0083	0.0090

**Table 10:** Information entropies of the cipher images and plain images, part 1.

Image	Channel	Proposed Algorithm Image a	Method [10] Image a	Method [24] Image a	Proposed Algorithm Image d	Method [10] Image d	Method [24] Image d
Plainimage	R	6.9581	6.9581	6.9581	7.7047	7.7047	7.7047
	G	6.8945	6.8945	6.8945	7.4724	7.4724	7.4724
	B	6.1365	6.1365	6.1365	7.7502	7.7502	7.7502
	RGB	7.2528	7.2528	7.2528	7.7604	7.7604	7.7604
Cipherimage	R	7.9992	7.9991	7.9992	7.9991	7.9991	7.9988
	G	7.9993	7.9989	7.9992	7.9991	7.9991	7.9992
	B	7.9993	7.9988	7.9993	7.9991	7.9990	7.9989
	RGB	7.9997	7.9996	7.9997	7.9996	7.9995	7.9996

**Table 11:** Information entropies of the cipher images and plain images, part 2.

Image	Channel	Proposed Algorithm Image g	Method [10] Image g	Method [24] Image g	Proposed Algorithm Image j	Method [10] Image j	Method [24] Image j
Plainimage	R	6.9571	6.9571	6.9571	7.7067	7.7067	7.7067
	G	6.8955	6.8955	6.8955	7.4734	7.4734	7.4734
	B	6.1355	6.1355	6.1355	7.7512	7.7512	7.7512
	RGB	7.2548	7.2548	7.2548	7.7614	7.7614	7.7614
Cipherimage	R	7.9982	7.9981	7.9982	7.9993	7.9993	7.9989
	G	7.9983	7.9979	7.9982	7.9995	7.9995	7.9994
	B	7.9994	7.9985	7.9994	7.9981	7.9980	7.9979
	RGB	7.9996	7.9995	7.9996	7.9994	7.9993	7.9994

**Table 12:** NPCRs and UACIs at different positions (%), Part 1.

Position	Proposed Algorithm Position (12, 34)	Method [10] Position (12, 34)	Method [24] Position (12, 34)	Proposed Algorithm Position (34, 56)	Method [10] Position (34, 56)	Method [24] Position (34, 56)
NPCR	99.6232	99.6222	99.6218	99.6215	99.5015	99.6187
UACI	33.4574	33.4504	33.4767	33.4952	33.3952	33.4850

**Table 13:** NPCRs and UACIs at different positions (%), Part 2.

Position	Proposed Algorithm Position (56, 78)	Method [10] Position (56, 78)	Method [24] Position (56, 78)	Proposed Algorithm Position (78, 90)	Method [10] Position (78, 90)	Method [24] Position (78, 90)
NPCR	99.6170	99.4170	99.6123	99.5971	99.1971	99.6223
UACI	33.5326	33.2326	33.3979	33.4755	33.3755	33.4759

**Table 14:** Comparison of the proposed algorithm’s key space with other algorithms.

Algorithm	Proposed	[9]	[12]	[24]
Key space	$10^{56}\times 2^{128}$	$2^{192}$	$2^{128}$	$2^{256}$
